# Laser Speckle Rheology for evaluating the viscoelastic properties of hydrogel scaffolds

**DOI:** 10.1038/srep37949

**Published:** 2016-12-01

**Authors:** Zeinab Hajjarian, Hadi Tavakoli Nia, Shawn Ahn, Alan J. Grodzinsky, Rakesh K. Jain, Seemantini K. Nadkarni

**Affiliations:** 1 Wellman Center for Photomedicine, Massachusetts General Hospital, Harvard Medical School, Boston, MA, USA; 2 Edwin Steele Laboratory for Tumor Biology, Massachusetts General Hospital, Harvard Medical School, Boston, MA, USA; 3 Department of Electrical and Computer Engineering, University of Illinois at Urbana-Champaign, Urbana, IL, USA; 4 Department of Mechanical Engineering, Massachusetts Institute of Technology, Cambridge, MA, USA; 5 Department of Biological Engineering, Massachusetts Institute of Technology, Cambridge, MA, USA; 6 Department of Electrical Engineering, Massachusetts Institute of Technology, Cambridge, MA, USA.

## Abstract

Natural and synthetic hydrogel scaffolds exhibit distinct viscoelastic properties at various length scales and deformation rates. Laser Speckle Rheology (LSR) offers a novel, non-contact optical approach for evaluating the frequency-dependent viscoelastic properties of hydrogels. In LSR, a coherent laser beam illuminates the specimen and a high-speed camera acquires the time-varying speckle images. Cross-correlation analysis of frames returns the speckle intensity autocorrelation function, *g*_*2*_(*t*), from which the frequency-dependent viscoelastic modulus, G*(*ω*), is deduced. Here, we establish the capability of LSR for evaluating the viscoelastic properties of hydrogels over a large range of moduli, using conventional mechanical rheometry and atomic force microscopy (AFM)-based indentation as reference-standards. Results demonstrate a strong correlation between |G*(*ω*)*|* values measured by LSR and mechanical rheometry (*r* = *0.95, p* < *10*^*−9*^), and z-test analysis reports that moduli values measured by the two methods are identical (p > 0.08) over a large range (47 Pa – 36 kPa). In addition, |G*(*ω*)*|* values measured by LSR correlate well with indentation moduli, *E*, reported by AFM (*r* = *0.92, p* < *10*^*−7*^). Further, spatially-resolved moduli measurements in micro-patterned substrates demonstrate that LSR combines the strengths of conventional rheology and micro-indentation in assessing hydrogel viscoelastic properties at multiple frequencies and small length-scales.

Biomaterials and hydrogels are increasingly used in tissue engineering, regenerative medicine, drug-delivery, and mechanobiology research owing to their unique biocompatibility, tunable compliance, deformability and stress resilience^1^,^2^,^3^,^4^,^5^,^6^. To fully integrate into biological systems, these biomimetic scaffolds exhibit distinct mechanical properties, similar to natural tissues at potential sites of implantation. Due to their large water content, tissues and hydrogels are viscoelastic, exhibiting both solid-like and liquid-like traits at different deformation rates and length-scales. This complex mechanical behavior is best defined by the frequency-dependent shear viscoelastic modulus, 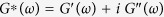. Here *G*′(*ω*) and *G*″(*ω*) are the elastic and viscous moduli, representing the solid-like and fluid-like features respectively and *ω* is the deformation frequency^7^. The macro-scale viscoelastic properties of tissues and biomimetic scaffolds enables them to withstand physiological and hemodynamic loads, yet exhibit sufficient flexibility. The micro-scale properties, on the other hand, influence the mechanical support provided to cells, impart mechanical cues to direct cellular growth and differentiation, control the diffusion of oxygen and nutrients, and regulate the release of bio-molecules and drugs^5^,^6^. Thus, there is a need to quantify the viscoelastic properties of tissue scaffolds and biomimetic gels in their native state at multiple length-scales and deformation frequencies in a non-destructive fashion without contact or sample manipulation.

We have previously described the Laser speckle rheology (LSR) approach for providing an index of tissue viscoelasticity *in situ* by measuring the time constant of laser speckle intensity fluctuations^8^,^9^,^10^,^11^. In LSR, a coherent laser beam illuminates the sample and light rays are scattered multiple times by endogenous particles within the specimen. A high-speed CMOS camera then collects the back-scattered light^8^,^9^,^10^,^11^,^12^. Constructive and destructive interference of rays at the CMOS sensor creates a fluctuating grainy intensity pattern, termed speckle. Speckle fluctuations are highly sensitive to Brownian displacements of scattering particles, in turn influenced by the viscoelasticity of the microenvironment and are analyzed via the speckle intensity autocorrelation curve, *g*_*2*_(*t*)^8^,^9^,^10^,^11^,^12^. In compliant samples, intrinsic particles undergo extensive Brownian excursions and frequently modify the trajectories of photons scattered from the sample to induce rapidly fluctuating speckle patterns. Rigid substrates, on the other hand, restrict Brownian movements and elicit only minute speckle modulations^8^,^10^,^11^. Our past reports on LSR and other micro-rheology studies have shown that G*(*ω*) can be successfully extracted by measuring the mean square displacements (MSD) of Brownian particles in soft homogeneous materials of low viscoelastic moduli such as colloids, gels, polymer solutions, and bio-fluids with known optical properties^13^,^14^,^15^,^16^,^17^,^18^,^19^,^20^. These prior studies have been limited to highly compliant or soft materials with viscoelastic moduli below a few kPa^15^.

In this paper, we detail and validate the LSR framework for quantifying the frequency-dependent viscoelastic behavior of hydrogels exhibiting an extended range of viscoelastic moduli significantly larger than prior studies^15^. To this end, we first prepare homogeneous agarose, polyacrylamide (PA), and polyethylene glycol di-acrylate (PEGDA) hydrogels with varied optical properties and scattering particle size distributions, spanning a large range of moduli (47 Pa–36 kPa), pertinent to natural and synthetic tissues. Despite the large water content within low density polymer network, these hydrogels are markedly more viscoelastic than primarily viscous silicone colloids, lipid emulsions, and hydrophobic silicone-based PDMS polymers, previously evaluated by LSR^13^,^14^,^15^. LSR measurements of the frequency-dependent |G*(*ω*)| curves of the hydrogels are compared with the results of rotational rheometry, the conventional standard for evaluating the bulk G*(*ω*). While mechanical rheometry provides information on the viscoelastic behavior of a sample at multiple oscillation frequencies, it fails to assess the local viscoelastic heterogeneities at the microscale^21^. The commercially available standard for probing the local mechanical properties at small scales is the AFM-based indentation. Nonetheless, the indentation modulus, *E*, evaluated by AFM, represents solely the elastic behavior at a single indentation rate, and does not fully reflect the frequency-dependent viscoelasticity needed for characterizing viscoelastic hydrogel and biomimetic scaffolds^22^,^23^,^24^. The LSR approach described here aims to bridge the strengths of both of the above conventional techniques by providing frequency-dependent measurements of viscoelastic moduli |G*(*ω*)| akin to mechanical rheometry yet with high spatial resolution similar to AFM. To fully examine the capacity of LSR for evaluating the frequency-dependent |G*(*ω*)| at micro-scale, we also fabricate composite PDMS-PEGDA substrates, exhibiting micro-patterned features of distinct viscoelastic properties. Spatially-resolved 2D maps of |G*(*ω*)| across the micro-patterned substrates are evaluated by LSR and compared to the conventional rheology measurements of the PDMS and PEGDA components. The results detailed below establish that LSR encompasses the desired traits of both conventional rheology and micro-indentation techniques, and evaluates the |G*(*ω*)|, at multiple frequencies and length-scales, without requiring sample contact or manipulation.

## Results

### Homogenous viscoelastic hydrogels

Agarose (0.5–3% v/w, N = 6), Polyacrylamide (PA) (3–20% Acrylamide (A)- 0.05–2.5% Bis-acrylamide (B), N = 6), and polyethylene glycol di-acrylate (PEGDA) (6–15% v/w, N = 6) hydrogels, covering a wide range of viscoelastic moduli (G*: 47–36 k Pa, @ *ω* = 1 Hz), were prepared. These gels were selected due to their widespread use in basic cellular mechanobiology, translational tissue engineering, and drug delivery research^25^,^26^,^27^. The choice of G* range was based on values reported for various biomimetic scaffolds^5^,^28^,^29^,^30^. To resemble scattering tissues, intralipid (Lyposyn^TM^ III, 10%, Hospira, IL) was added at 1% w/v to all specimens.

### Laser speckle rheology of homogeneous hydrogels

**Figure 1:**
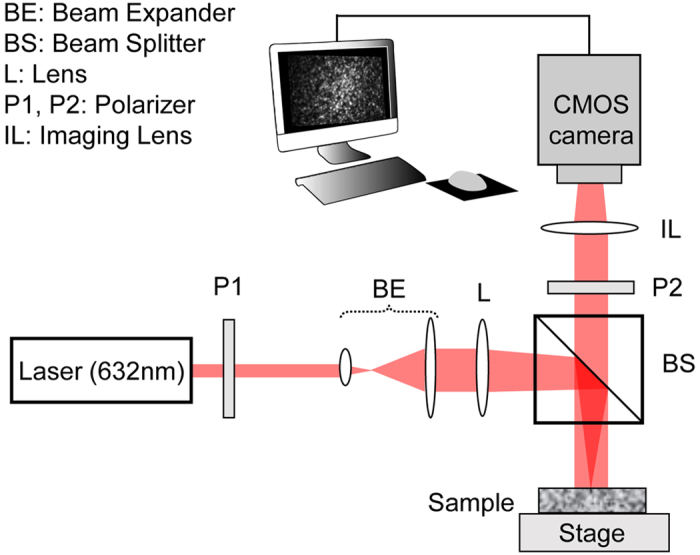
LSR optical setup. A randomly polarized laser beam is directed through a linear polarizer (P1) and collimated by a beam expander (BE). A lens (L) and a 50:50 beam splitter (BS) bring the beam to a 50 μm focal spot on the sample surface. Time series of both parallel and cross-polarized, back scattered speckle patterns are captured by the high-speed CMOS camera, through a polarizing filter (P2) and an imaging lens (L2). The iris within the imaging lens adjusts the pixel to speckle ratio.

**Figure 2:**
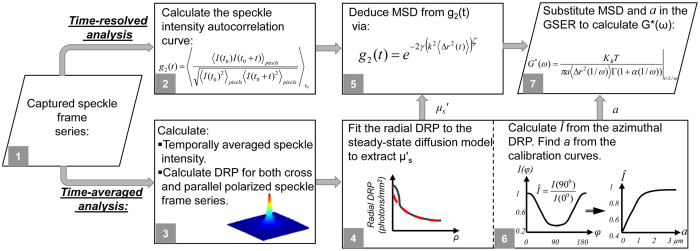
Flow-chart of LSR processing algorithm. **Box 1:** Speckle images are acquired at both parallel and perpendicular polarization states. **Box 2:** Cross-correlating the first speckle frame with subsequent frames returns the speckle intensity auto-correlation function, *g*_*2*_(*t*). **Box 3:** Temporal averaging of speckle frames yields the diffuse reflectance profile (DRP) at both parallel and cross polarization states. **Box 4:** The *μ*_*s*_′ is experimentally evaluated via curve-fitting to the radial cross-polarized DRP. **Box 5:** Optical properties determine the *γ* and *ζ* parameters in *g*_*2*_(*t*) = *exp*(−*2γ (k*^*2*^ < *Δr*^*2*^(*t*)*>*)^*ζ*^) and enable deducing the MSD. **Box 6:** The ratio of parallel-polarized DRP along short and long axes, i.e. *Î* = *I*(*φ* = *90*^*o*^)/*I*(*φ* = *0*^*o*^), is compared with a calibration curve to evaluate the average radius of scattering particles, *a*. **Box 7:** *MSD* and *a* are substituted in GSER to calculate the G^*^(*ω*).

**Figure 3:**
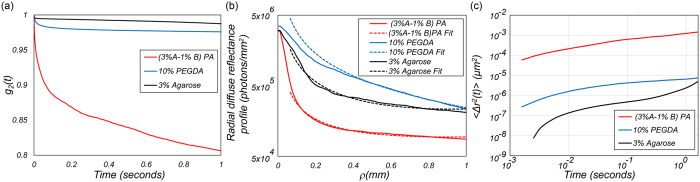
(**a**) Speckle intensity autocorrelation curves, *g*_*2*_(*t*), for (3% A-1% B) PA, 10% PEGDA, and 3% agarose. (**b**) The radial diffuse reflectance profile of the representative gels, obtained from temporally averaged speckle frame series (solid lines). A model function, based on diffusion theory is fitted to the DRP curves (dashed lines) to evaluate the sample optical properties. (**c**) The mean square displacements (*MSD*) of intralipid particles within the (3% A-1% B)PA, 10% PEGDA, and 3% agarose gels, obtained by replacing the *g*_*2*_(*t*) curves of Fig. 3(a) and the optical properties of the gels in the equation, derived from CT-MCRT.

The LSR optical setup and processing scheme flowchart are depicted in Figs 1 and 2, and elaborated in Methods^8^,^9^,^10^,^12^. Briefly, polarized Helium-Neon laser (632 nm) was focused on the sample and the back-reflected speckle patterns were imaged by a high-speed CMOS camera through a polarizer and an imaging lens. Speckle frames of 510 × 510 pixels were acquired at 739 frames per seconds (fps), for over 5 seconds at several points across the samples. Temporal cross-correlation analysis of speckle frames returned the speckle intensity autocorrelation function, *g*_*2*_(*t*) (Fig. 2, box 2)^8^,^9^,^11^,^12^,^13^,^14^,^15^. Figure 3a displays the *g*_*2*_(*t*) curves for three representative gels, namely (3% A-1% B) PA, 10% PEGDA, and 3% agarose. Clearly, the curve corresponding to (3% A-1% B) PA decayed considerably faster than 10% PEGDA and 3% agarose and plateaued at much lower value. In contrast, the *g*_*2*_(*t*) curve of 3% agarose decayed very slowly and plateaued at a significantly higher level. Thus, Brownian dynamics seemed to be much faster in (3% A-1% B) PA, indicating that this gel was the softest, with the lowest modulus. In contrast, particle trajectories were highly restricted in 3% agarose, implying that this gel was the most rigid one. We have previously reported that variations in optical properties could modify the *g*_*2*_(*t*) curves independent of sample viscoelasticity, and may lead to inaccurate MSD quantification^13^,^14^. This is because the rate of speckle fluctuations is modulated by both the extent of particle displacements, and the number of scattering events encountered in optical paths. Our prior studies demonstrated that in specimens of similar viscoelastic properties, the number of scattering interactions was proportionate to the reduced scattering coefficients *μ*_*s*_′^13^,^14^,^15^. Thus, *g*_*2*_(*t*) curves decayed more rapidly when *μ*_*s*_′ increased^13^,^14^,^15^. In contrast, number of scattering particles implicated in returning rays decreased with absorption coefficient, *μ*_*a*_, leading to slowly decorrelating *g*_*2*_(*t*) curves^13^,^14^,^15^. Therefore, to precisely evaluate the viscoelastic properties it was necessary to quantify and compensate for the influence of *μ*_*a*_ and *μ*_*s*_′. Using recently reported methods, we time-averaged the cross-polarized speckle images and measured the diffuse reflectance profile (DRP) as a function of distance from the illumination spot^13^,^15^. Assuming *μ*_*a*_*~0,* we then fitted a model obtained from light diffusion approximation to the radial DRP curve and experimentally calculated the *μ*_*s*_′ (Fig. 2, boxes 3 and 4)^13^,^31^. The radial DRP of (3% A-1% B) PA, 10% PEGDA, and 3% agarose are shown in Fig. 3b along with the fitted curves, revealing distinct *μ*_*s*_′ values of 0.9, 13.3, and 4.3 mm^−1^ respectively. Despite identical intralipid concentrations in all gels, differences in *μ*_*s*_′ were likely due to variations in refractive index mismatch and curing protocols, as discussed later^32^.

**Figure 4:**
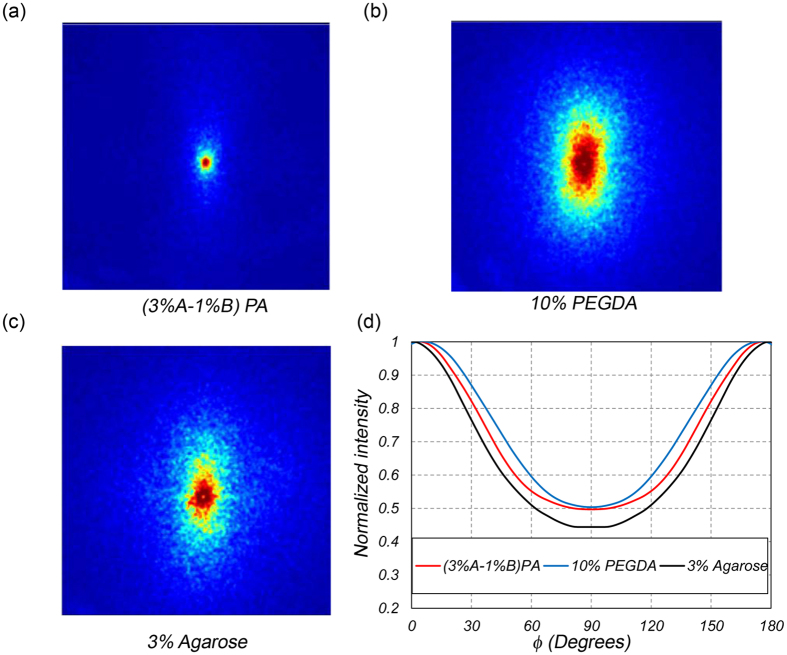
Diffuse reflectance profile (DRP) of (**a**) (3%-A, 1%-B) PA, (**b**) 10% PEGDA, and (**c**) 3% agarose, obtained from temporally averaged speckle frame series, collected in parallel-polarized state with respect to illumination beam. (**d**) Normalized DRP values vs. azimuth angle for all three gels.

Given *μ*_*a*_ and *μ*_*s*_′, the *g*_*2*_(*t*) curves were uniquely expressed in terms of the MSD of Brownian particles (Fig. 2, box 5). We have previously developed a modified equation to derive the MSD values, <*Δr*^*2*^(*t*)>, from *g*_*2*_(*t*) in moderately scattering samples of negligible absorption, as: *g*_*2*_(*t*) = *exp*(−*2γ (k*^*2*^ < *Δr*^*2*^(*t*)>)^*ζ*^). Here *γ and ζ* were empirical variables, related to *μ*_*s*_′, and *k* was the wavenumber^13^,^15^. Figure 3c displays the MSD of intralipid particles within the representative gels. Extended MSD trajectories were observed in the (3% A-1% B) PA, as opposed to 10% PEGDA and 3% agarose specimens. The acute sensitivity of LSR to particle displacements of a few angstroms was evident within the highly viscoelastic 3% agarose. Unlike a simple linear trend in purely viscous fluids^33^, the MSD curves of Fig. 2c, exhibited non-trivial behaviors, reflecting the complex viscoelastic nature of the microenvironment. Since for given viscoelastic and optical properties, smaller intrinsic scattering particles had greater MSD trajectories, the average size, *a*, of light scattering particles had to be estimated to deduce the absolute magnitude of G*(*ω*)^15^. We recently developed a straightforward approach for evaluating the average radii of scattering particles, *a*, from the speckle frame series (Fig. 2, box 6)^15^. Briefly, co-polarized speckle images were temporally averaged and converted to relative DRP as a function of azimuth-angle^15^. We then calculated the ratio of DRP across short and long axes, i.e. *Î* = *I*(*φ* = *90°*)*/I*(*φ* = *0°*), and compared it with a calibration curve to identify the corresponding *a*^15^. Figure 4a–d show co-polarized DRP images and the relative DRP vs. azimuth angle for (3% A-1% B) PA, 10% PEGDA, and 3% agarose. The *Î* values were 0.48, 0.46, and 0.45, respectively, corresponding to *a*~100 nm for all gels, consistent with previous reports on the particle size distribution of intralipid, as discussed later^15^,^34^. The *MSD* evaluated above and *a* = 100 nm were then replaced in the generalized Stokes-Einstein relation (GSER) to derive G*(*ω*) as (Fig. 2, box 7)^13^,^14^,^19^:


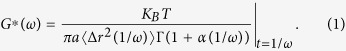


Here, *K*_*B*_ is the Boltzman constant, *T* is the temperature (Kelvins), *α* is the log-log slope of *MSD* at *t* = *1/ω*, and *Γ* is the gamma function^19^.

### Comparison with mechanical rheometry

**Figure 5:**
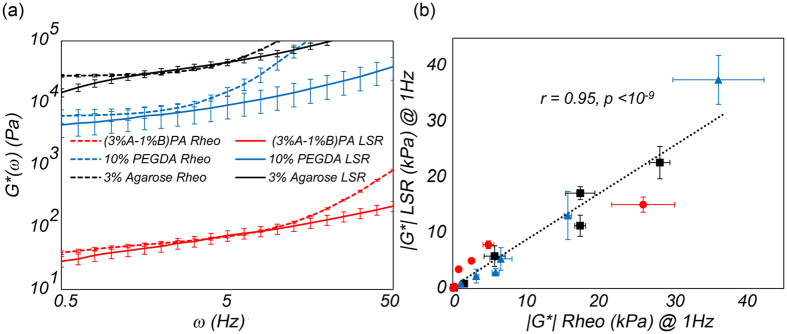
(**a**) The complex viscoelastic modulus, G*(*ω*), curves obtained from LSR (solid lines) and mechanical rheometry (dashed line) for (3% A-1% B) PA, 10% PEGDA, and 3% agarose. Close correspondence is observed between the two measurements over the frequency range of 0.5–10 Hz. Deviation at higher frequencies are due to emergence of inertial effects in conventional rheology, which makes the results unreliable. Divergences at frequencies below 0.1 Hz are caused by the significant influence of compressional, rather than shear fluctuations of the hydrogel network, on the slow Brownian dynamics at these frequencies (**b**) Scatter diagram of |G*(*ω*)*|* evaluated at 1 Hz obtained from LSR and conventional rheology for all the gels (N = 18, PA gels: red circles, PEGDA gels: blue triangles, agarose gels: black squares). A strong, statistically significant correlation is observed between the two measurements over the moduli range of 47 m Pa–36 kPa (r = 0.95, p < 10^−9^). Z-test analysis confirmed that the difference between LSR and rheometry measurements is insignificant (p = 0.08).

LSR measurements of G*(*ω*) in homogeneous hydrogels were compared with mechanical rheometry results. Mechanical rheometry is a traditional standard for evaluating bulk G*(*ω*), by destructively shearing the specimen between two plates and calculating the stress to strain ratio at few deformation frequencies. Figure 5a displays the G*(*ω*) curves obtained from LSR (solid lines) compared with those evaluated by a mechanical rheometer (ARG2, TA Instruments, DE, dashed lines) for the 3 representative hydrogels. Similar to rheometry, LSR accurately detected the relative differences in the viscoelastic compliance of the hydrogels. Furthermore, the absolute magnitudes of G*(*ω*), obtained from LSR, closely agreed with mechanical rheometry, particularly within 0.5–10 Hz range. For instance, G*(*ω*) values measured by LSR at 1 Hz for (3% A-1% B) PA, 10% PEGDA, and 3% agarose were: 39 ± 7 Pa, 5.3 ± 2 kPa, and 22.6 ± 2.9 kPa, respectively. For the same gels, mechanical rheometry reported similar values of 47 ± 2 Pa, 6.5 ± 1.5 kPa, and 28.1 ± 1.3 kPa, at 1 Hz. Above 10 Hz mechanical rheometry became unreliable due to tool inertia, as discussed later^13^,^21^,^35^. This was more evident in 10% PEGDA and 3% agarose gels, for which we used a smaller rheometer top plate (8 mm dia.). Using a larger 40 mm tool for mechanical rheometry of (3% A-1% B) PA increased the contact area and partially alleviated the inertial effect, as evidenced by the smaller deviations of rheometry measurements from the LSR results. At low frequencies (<0.5 Hz) the LSR-derived moduli deviated from mechanical rheometry because the slow Brownian dynamics at these frequencies were largely caused by compressional, but not shear fluctuations of the hydrogel network as discussed later^21^,^36^. Figure 5b depicts the scatter diagram of |G*| at 1 Hz evaluated by LSR and conventional rheology (N = 18). A strong, statistically significant correlation (r = 0.95, p < 10^−9^) was observed between LSR and mechanical rheometry across the whole moduli range from the softest hydrogel at 47 Pa ((3% A-1% B) PA) to the most rigid one at 36 kPa (15% PEGDA). Furthermore, z-test analyses indicated that differences between moduli measured by LSR were no different from those measured by mechanical rheometry (p ≥ 0.08), demonstrating the accuracy of LSR in quantifying the frequency-dependent G*(*ω*) over multiple decades of moduli from a few Pa to tens of kPa.

### Comparison with AFM-based indentation

**Figure 6:**
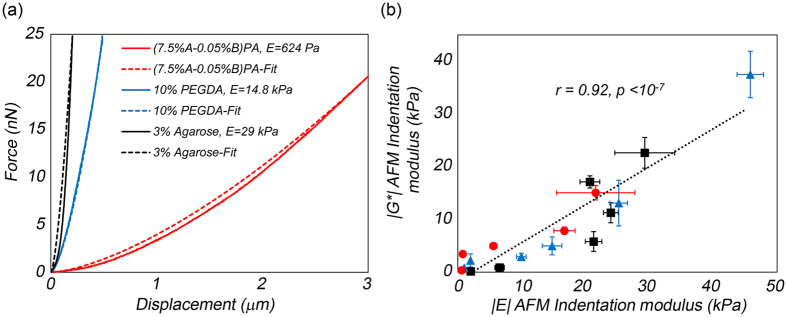
(**a**) Typical force-distance curves for the (7.5% A-0.05% B) PA, 10% PEGDA, and 3% agarose gels. The corresponding best fitted curves (red), obtained using the Hertzian model are also displayed (**b**) Scatter diagram of |G*(*ω*)*|* values at 1 Hz obtained from LSR and the indentation modulus, *E*, measured by AFM at the indentation rate of 2 μm/s for viscoelastic gels (N = 17, PA gels: red circles, PEGDA gels: blue triangles, agarose gels: black squares). Linear regression analysis declares a strong, statistically significant correlation (r = 0.92, p < 10^−7^) for *E:* 624 Pa–46 kPa.

The viscoelastic modulus at the micro-scale experienced by cells may not be identical to macro-scale properties of scaffolds. Thus, approaches that measure micro-scale mechanical properties provide unique advantages for cell-scaffold studies^24^,^37^,^38^,^39^. The current reference standard for measuring micro-scale properties is AFM-based indentation. Therefore, we further assessed the relationship between |G*(*ω*)*|* evaluated by LSR and micro-indentation moduli, *E*, evaluated by AFM-based indentation in the above hydrogels. To obtain AFM measurements, the specimens were indented by a small cantilevered-probe and *E* was calculated by fitting the Hertz model to the curve displaying the applied force versus cantilever displacement^22^,^23^,^24^,^40^. The (3% A-1% B) PA gel was too soft and adhesive for indentation and had to be excluded from the analysis. As such, AFM measurements were conducted on a total of 17 out of 18 prepared hydrogels. Figure 6a displays the typical force-displacement curves for (7.5% A-0.05% B) PA, 3% agarose, and 10% PEGDA gels at the approach rate of 2 μm/s. The rapidly increasing force in 3% agarose and 10% PEGDA, compared to (7.5% A-0.05% B) PA, pointed to the higher elastic moduli of these gels. The *E* was calculated as 624 ± 27 Pa, 14.8 ± 1.5 kPa, and 29.2 ± 4.7 kPa for (7.5% A-0.05% B) PA, 10% PEGDA, and 3% agarose, respectively. Figure 6b displays the scatter diagram of LSR-measured |G*(*ω*)*|* values at 1 Hz and *E* at 2 μm/s. A statistically significant correlation (r = 0.92, p < 10^−7^) was observed between the two measurements for *E*: 624 Pa–46 kPa, establishing that |G*(*ω*)*|* measured by LSR were closely related with *E* measured by AFM.

### Micro-mechanical mapping of G*(*ω*) using LSR

**Figure 7:**
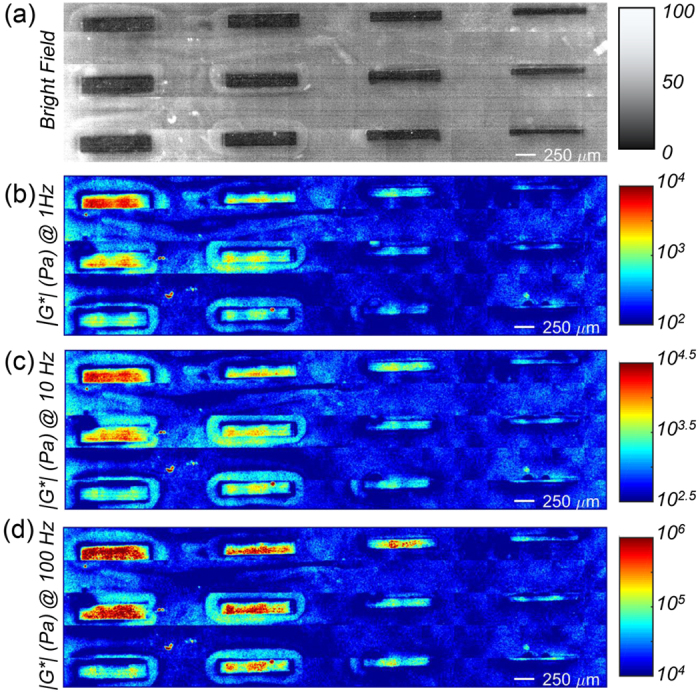
(**a**) Bright field image of the micro-fabricated composite PDMS-PEGDA phantom. A total of 12 PDMS bars are visible within the PEGDA background. The bars in successive columns are 1 mm long and 200, 150, 100, and 80 μm wide, respectively. (**b**) Spatially-resolved G*, evaluated at 1 Hz. In the color-bar, the moduli range of 100 Pa–10 kPa, are represented by blue to red hues. The 80 μm wide bars are barely visible at 1 Hz. (**c**) Spatially-resolved G*, evaluated at 10 Hz. In the color-bar, the moduli range of 300 Pa–300 kPa, are represented by blue to red hues. The 80 μm wide stiff bars are distinguished within the plain soft PEGDA 5% background. (**d**) Spatially-resolved G*, evaluated at 100 Hz. In the color-bar, the moduli range of 10 kPa–1 MPa, are represented by blue to red hues. Significant contrast is observed between stiff PDMS bars and the PEGDA 5% background at all length-scales.

We further examined the capability of LSR for merging the advantages of standard mechanical rheometry that measures frequency-dependent viscoelastic behavior with mechanical mapping of spatial heterogeneities at the micro-scale. Soft lithography techniques were used to construct substrates with heterogeneous viscoelastic features of tens of microns in size. A composite PDMS-PEGDA gel was microfabricated, featuring stiff PDMS bars of assorted widths surrounded by soft PEGDA 5% hydrogel. To enable micromechanical assessment of viscoelastic properties, the substrate was illuminated by an expanded beam and scanned at 450 μm steps in transverse direction. Speckle images were acquired at 250 fps, for 1 second, through an objective lens (10x, *NA* = 0.25, Olympus). Spatio-temporal processing of speckle frames returned the *g*_*2*_(*t*) curve for individual pixels from which spatially-resolved G*(*ω*) was deduced, as explained above. Figure 7a–d display the bright-field image and the 2D spatially-resolved LSR color-map of |G*(*ω*)*|* within the micro-patterned substrate at 1, 10, and 100 Hz. The PDMS bars in successive columns of Fig. 7a were 1 mm long, and 200, 150, 100, and 80 μm wide, respectively. The color-bars in Fig. 7b–d were scaled to provide the highest contrast at the corresponding frequencies. The regions of increased stiffness in G* maps coincided with PDMS bars in the bright field image, whereas softer regions corresponded to PEGDA 5% background. The increased stiffness of PEGDA 5% around the bars was likely due to drying, and closely mirrored similar features in the bright-field image. Comparison of Fig. 7b–d revealed that the G* map, evaluated at 100 Hz, exhibited the highest contrast and resolution. The modest resolution and contrast of G* color maps at low frequencies was due to reduced spatial and temporal averaging employed in calculating the pixel-wise *g*_*2*_(*t*) curves, at longer decorrelation times, which in turn limited the statistical accuracy of MSDs at longer times, and G* at lower frequencies (Supplementary Video V1). Comparison of mechanical rheometry measurements with G* color-maps yielded similar values: G* (@1 Hz) for PEGDA 5% was 275 ± 122 Pa by rheometry and 272 ± 0.9 Pa by LSR; and for PDMS was 10.8 ± 2.1 kPa by rheometry and 7 ± 0.03 kPa by LSR. From Fig. 7a–d, it was clear that LSR conveniently resolved even the smallest 80 μm-wide stiffer PDMS bars from the soft PEGDA 5% background. Moreover, heterogeneous moduli were identified within the bars, demonstrating the resolution and sensitivity of LSR to variations in G* at length scales of a few 10 *μm*. These heterogeneities were likely caused by variations in casting of PDMS elastomer within the soft background and the leakage of soft PEGDA gel to the PDMS compartments. We also constructed a test phantom, with smaller differences between the moduli of the bars and the background gel, composed of PEGDA 10% (G* = 6.5 ± 1.5 kPa @ 1 Hz) and PDMS (G*10.8 ± 2.1 kPa @ 1 Hz) (Supplementary Fig. S1). These gels were stiffer and hence easier to cast. Thus, the composite gel exhibited more defined borders and the bars appeared less heterogeneous. Moreover, since the difference between the moduli of the background and the bars was smaller, the contrast between the two sections was reduced. These results demonstrated the capability of LSR to render spatially heterogeneous, frequency-dependent viscoelastic properties of materials with micro-scale resolution and high sensitivity to moduli differences.

## Discussion

Here we detailed the LSR framework for measuring the viscoelastic properties of hydrogels with unknown optical properties and scattering sizes and tested its accuracy via comparison with standard mechanical rheometry and AFM-based indentation. We showed close agreement between LSR and rheometry, confirming that LSR accurately quantifies the complex frequency-dependent viscoelastic modulus, a metric representing both the viscous and elastic traits, over a wide range of deformation frequencies encountered in physiological processes and multiple decades of moduli. To establish the competence of LSR with the AFM-based indentation, the standard for micromechanical testing, we also demonstrated the correlation between the viscoelastic moduli, evaluated by LSR, and the indentation moduli, evaluated by AFM. Subsequently we showed that LSR resolves frequency-dependent viscoelastic moduli of micro-fabricated mechanical features in specialized gel substrates with high resolution and contrast. Taken together, these results proved that LSR merges the advantages of conventional rheometry for measuring frequency dependent viscoelastic behavior with the opportunity for micro-mechanical mapping afforded by AFM via a non-contact, all-optical approach.

We readily inferred the relative differences in viscoelastic properties of homogeneous hydrogels from the speckle fluctuations rates quantified by the *g*_*2*_(*t*) curves of Fig. 3a. The partially non-fluctuating speckle patterns of highly viscoelastic 3% agarose and 10% PEGDA were implicated in the higher plateau of *g*_*2*_(*t*) curves and revealed the substantial rigidity of the gel matrix entrapping intralipid particles. Still, to derive the bulk |G*(*ω*)|, LSR had to quantify and compensate for variations in optical properties^13^,^14^. We maintained similar intralipid concentrations (1% w/v) in the hydrogels, thus one would expect identical optical properties of *μ*_*a*_ = 0 and *μ*_*s*_′ ~ 1 mm^−1^. Yet, DRP curves of Fig. 3b, obtained from time-averaged speckle frames, revealed substantially different *μ*_*s*_′ values caused by variations in curing processes and constituent materials. For instance, we speculate that the partially demulsified intralipid particles in heated agarose solutions floated and re-coalesced to increase the *μ*_*s*_′ (4.3–17.5 mm^−1^) in these samples^32^,^41^. We also observed that the polymerization of low molecular weights (M_n_ = 575) PEGDA increased the refractive index mismatch between the infused water and the emerging gel meshwork, creating a turbid hydrogel with increased *μ*_*s*_′ (10.4–15.3 mm^−1^)^42^,^43^. In contrast, PA gels were transparent and the intralipid particles were evenly distributed in the polymer network, leading to *μ*_*s*_′ of 1 mm^−1^, as expected^19^,^44^,^45^,^46^.

Since the hydrogels in this study were primarily scattering, *μ*_*a*_ was assumed negligible, and only *μ*_*s*_′ was deduced from the logarithmic slope of DRP. This approach provided a reasonable approximation of the depth-integrated optical properties within the illuminated volume. In materials with heterogeneous and depth-varying scattering, the radially-resolved DRP may be measured to exploit photons remitted further away from the illumination spot that likely return from deeper regions^9^,^47^. Accordingly, spatially-resolved optical properties may be extracted, assuming that DRP follows a piecewise model, with the slope of segments at distinct distances from the illumination center reflecting the optical properties of different layers^9^,^47^. While in the current study, *μ*_*a*_*~0*, for samples with non-negligible *μ*_*a*_, such as in blood, we have previously shown that both *μ*_*a*_ and *μ*_*s*_′ can be estimated from the DRP^16^.

Next, we used a modified expression derived from our prior correlation-transfer Monte-Carlo ray tracing methods, to deduce the MSD curves of Fig. 3c (see Methods)^13^,^15^. These curves were neither linearly growing as in purely viscous fluids nor flat similar to elastic solids, and represented the more complex viscoelastic behavior of hydrogels. For these porous gel that incorporated particles, the overall MSD was estimated to be a weighted average of the displacements of particles within the pores as well as the fibers. The MSD of probe particles at early time scales, was primarily diffusive and reflected the local hydrodynamics of the pores, whereas at longer times it converged to the MSD of fibers, which portrayed the bulk viscoelasticity of the gel meshwork^48^. In such complex realistic gels, the MSD exhibited multiple relaxation times, commensurate with the structure of the gel^48^.

Consequently, knowledge of scattering particle size was important for deducing the G*(*ω*) from the MSD (Eqn. 1). The intralipid emulsions have a well-established size distribution^32^,^34^,^49^. Published reports using dynamic light scattering (DLS) and electron microscopy studies estimate a mean radius of *a* ~ 100 nm for intralipid particles^32^,^34^,^50^. We exploited a DLS-based particle sizer (ZetaSizer, Malvern Instruments, UK) to confirm that *a* = 100 nm. Our experimental derivation of *a* = 100 nm from the co-polarized DRP patterns (Fig. 2, box 6, and Fig. 4) matched these results.

The bulk G*(*ω*) of homogeneous hydrogels was obtained through replacing the *MSD* and *a* in Eqn (1). We observed a close agreement between LSR and conventional rheometry measurements at 0.5–10 Hz frequencies and over moduli range of 47 Pa–36 kPa^51^. LSR results were derived via Brownian dynamics induced by both shear and compressional thermal fluctuations. Compressional fluctuations dominated at low frequencies, causing LSR measurements to deviate from shear-based mechanical rheometry^21^,^36^. This low frequency limit depended on the viscoelastic susceptibility and the microstructure of the specific material and varied from 0.01–0.1 Hz for primarily viscous biofluids, investigated in our prior work, to 0.1 Hz, for hydrogels evaluated here (Fig. 5a)^21^,^51^,^52^. The upper frequency limit however was reached when inertial effects prevented sufficient straining of the specimen. In mechanical rheometry, at frequencies above 10 Hz, the strain waves generated by rheometer tool failed to penetrate across the gap between the parallel plates of the rheometer tool and merely sheared the sample surface, overestimating the high-frequency modulus^13^. On the contrary, in LSR the inertial effects only dominated when the penetration depth of shear waves became comparable to the scattering particle size. Therefore, the sub-micron sized scattering particles extended the onset of inertial effects beyond several hundreds of kHz, opening a new window of frequencies inaccessible to mechanical rheometry^21^,^35^,^51^,^52^. The practical upper limit was further tied to the camera frame rate. In the current study, a frame rate of 739 fps limited the highest measureable frequency to below 100 Hz. By employing acquisition speeds of a few 100 kfps, higher frequencies, in the order of 10^5^ Hz may be achieved.

Besides spanning a wide range of frequencies, LSR measured a considerable extent of moduli. We previously showed that LSR accurately quantified the moduli of extremely soft biofluids (few mPa)^13^,^15^,^16^. The size of scattering particles, *a*, the ability to resolve their infinitesimal motions, *δ*_*r*_, and the thermal energy, *K*_*B*_*T*, set the upper limit of moduli accessible to LSR to: *K*_*B*_*T/*(*δ*_*r*_^*2*^*a*). We clarified that the highly sensitive multi-speckle detection enabled resolving displacements of *δ*_*r*_∼*Å*, as seen in Fig. 3c, leading to an upper limit of tens of *kPa*. For predominantly elastic materials, however, the nearly frozen speckle potentially returns a *g*_*2*_(*t*) curve that plateaus at unity and an MSD that flattens to zero, implying that thermal energy is inadequate for provoking a detectable displacement. Alternatively, intrinsic thermal fluctuations could be complemented with external stress fields to elicit detectable strains in considerably stiffer materials^21^. The strong, statistically significant correlation between LSR and rheometry in hydrogels of unknown optical properties and particle sizes, observed in Fig. 5a and b, paves the path for biomechanical evaluation of natural and synthetic tissue scaffolds with assorted composition, morphology, and microstructure.

Because our metric of interest is the viscoelastic modulus, here we primarily focused on establishing the ability of LSR to accurately quantify the G*. Nevertheless, since the only commercially available standard for micro-scale mechanical testing is AFM, we also investigated the correlation between LSR and AFM measurements. The high correspondence between |G*| measured by LSR at 1 Hz, and the indentation modulus, *E*, evaluated by AFM (Fig. 6b) suggested that LSR closely mirrors the micro-scale stress-strain measurements. Using various indentation rates in AFM could partially elucidate the frequency-dependence of *E*. However, the commercial AFM fell short in quantifying the complex, dynamic |E*| (similar to G* in LSR) unless coupled with a high-frequency actuator^39^. Still, the contact-based AFM was not conducive to mechanical evaluation of soft and adhesive specimens, such as the (3% A-1% B) PA. In contrast, the non-contact nature of LSR permitted for evaluating adhesive and highly compliant materials, not easily assessed by AFM^16^,^53^.

Spatially-resolved LSR measurements in the micro-fabricated phantom established the capacity of this new tool for evaluating the viscoelastic compliance at multiple frequencies and length scales to accommodate probing local mechanical heterogeneities (Fig. 7). In the current LSR system, using a 10X, 0.25 *NA* objective, set the speckle grain size to 1.5 *μm* (airy disk) which was imaged on a 3 × 3 pixel array (pixel/speckle = 9). Using a 25 × 25 Gaussian window for calculating the spatially-resolved *g*_*2*_(*t*) curves, the calculated lateral resolution for G* mapping (Fig. 7) was equal to 12.5 *μm*. Through serpentine scanning and high-speed image acquisition, LSR could survey a 1 cm^2^ area, with resolution of 12.5 *μm* within 20 minutes. AFM can potentially evaluate a map of static *E* through scanning the sample surface in steps and acquiring force-displacement curve at each location. However, obtaining each curve takes a few seconds, which would result in measurement times of several hours to evaluate a similar 1 cm^2^ area^37^. Moreover, the contact-based, invasive, and manipulative nature of AFM restrict the possibility of evaluating mechanical properties of cell-culture systems, under sterile conditions. Furthermore, we have previously shown that due to the susceptibility of *g*_*2*_(*t*) curves to sub-wavelength scattering particle displacements, LSR is sensitive to moduli changes as small as ΔG* = 0.5 Pa^16^. Thus, LSR may likely be used to evaluate dynamic changes that occur due to ECM remodeling in biomimetic systems with high measurement sensitivity. Increasing the CMOS sensor bit depth from 8 to 16, acquiring more speckle spots, and collecting longer optical paths susceptible to rapid decorrelations could improve the LSR sensitivity to intensity fluctuations and slightly extend its dynamic range^11^. Given its high spatial resolution and multi-frequency measurement capabilities, LSR provides a unique approach for investigating and developing synthetic tissue scaffolds. It also provides biologists with a new imaging tool to address key questions concerning the mechanosensitive regulation of cell morphology, physiology, and behavior by ECM components^54^,^55^,^56^.

## Methods

### Homogeneous viscoelastic hydrogel preparation

We made three sets of hydrogels, exhibiting a wide range of viscoelastic properties pertinent to tissues and biomaterials. Agarose gels of 0.5%, 1%, 1.5%, 2%, 2.5%, and 3% concentrations were prepared by sprinkling low gelling-point agarose powder (100 mg per 1%, Sigma-Aldrich, MO) into beakers containing 9 ml of deionized water and magnetic beads stirring at 500 rpm. The weights of beakers were recorded before heating. The solutions were covered and brought to boil, stirring continuously to prevent clumping, until appeared transparent. Hot deionized water was used to maintain the initial weights of solutions. Finally, 1 ml of intralipid was added, the mixtures were poured in 35 mm dia. petri dishes and left to cure at room temperature. The shear moduli of agarose gels ranged between 162 Pa–28 kPa at ω = 1 Hz.

Polyethylene glycol de-acrylate (PEGDA) gels were prepared by making 9 ml PEGDA solutions at 6%, 8%, 9%, 10%, 12%, and 15% concentrations (Sigma-Aldrich, molecular weight: M_n_ 575) in phosphate buffer saline (PBS). Solutions were completed with 1 ml of intralipid and 1% w/v of photo-initiator (DAROCUR 1173, Ciba Specialty Chemical, Switzerland). About 200 μl of each solution was pipetted into an imaging chamber (dia.: 9 mm, depth: 2 mm, Grace Bio-Labs, OR) featuring a clear polycarbonate window. The chamber was placed within 1 cm of a UV curing system (beam dia. 12 mm, λ = 365 nm, 175 mW/cm^2^, Thorlabs, NJ) and illuminated for 3 minutes. The G* of PEGDA gels ranged between 1 kPa–36 kPa at ω = 1 Hz.

Polyacrylamide gels were prepared following standard protocols^28^. Briefly, the required volumes of acrylamide and bis-acrylamide in a 5 ml final precursor mixture were calculated based on the concentrations of available stock solutions, i.e. 40% acrylamide and 2% bis-acrylamide (Sigma-Aldrich), and the final desired concentration pairs of (3%, 1%), (7.5%, 0.05%), (7.5%, 0.2%), (7.5%, 0.6%), (10%, 2%), and (20%, 2.5%). Subsequently, the solutions were brought to 5 ml volume by adding 500 μl of intralipid, 1250 μl of Tris-HCl buffer (pH 8.8), and deionized water, followed by 25 μl of 10% ammonium persulfate (APS) and 5 μl of Tetramethylethylenediamine (TEMED) (Sigma-Aldrich) to initiate and catalyze the polymerization. The shear moduli of PA gels ranged between 150 Pa–30 kPa at *ω* = 1 Hz.

### Micro-fabricated composite PDMS-PEGDA substrates preparation

We used established methods to create the micro-fabricated phantom^26^,^45^,^57^. A photomask, featuring bars of assorted widths (i.e. 250, 200, 150, 100, 80, 60, 50, 30, and 10 μm) was sketched in Solidworks (DS SolidWorks, MA) and printed at high resolution (CAD/Art Services, OR). To create the mold, a 5″ silicon wafer was solvent-cleaned and plasma-treated to remove residues (Technics 500*-*II Plasma Etcher). A 400 μm-thick layer of SU8-2100 photo-resist (MicroChem, MA) was spin-coated on the wafer (Headway, TX). To engrave the patterns, the wafer was exposed to UV through the photomask within a mask-aligner (MJB3, SUSS MicroTech, Germany), and developed. To prepare the PDMS, resin and curing agent (Sylgard^®^ 184 silicone elastomer, Dow Corning, Belgium) were mixed in 10:1 ratio. To differentiate PDMS from PEGDA, carbon powder (430 nm dia., Sigma Aldrich) was added in 0.5% concentration, prior to centrifugal mixing (THINKY ARE-250, Japan). The PDMS was slowly poured on the mold, degassed in a vacuum chamber, and cured for 2 hours at 60 °C. It was then gently peeled off and cut into blocks. Protruded bars on the PDMS bonded to a glass coverslip by plasma treatment. PEGDA 5% and 10% solutions were drawn to the spacing between PDMS and glass via capillary action and cured by UV illumination. The final micro-fabricated phantoms featured stiff PDMS bars (G* = 10.8 ± 2.1 kPa @1 Hz) in a soft PEGDA 5% (G* = @ 275 ± 122 Pa @ 1 Hz) as well as moderately stiff PEGDA 10% (G* = @ 6.5 ± 1.5 kPa @ 1 Hz) backgrounds.

### Laser Speckle Rheology Testing

The LSR optical setup is displayed in Fig. 1. Briefly, light from a randomly-polarized Helium-Neon laser (632 nm, 45 mW, JDSU, CA) was polarized, expanded (10x), and focused by a lens and a 50:50 beam-splitter to a 50 *μ*m spot on the sample. Focused beam illumination enabled evaluating the diffuse reflectance profile (DRP) of the specimens. The light scattered off the sample was collected at 180^o^ back-scattering geometry through a linear polarizer and an imaging lens (MLH-10x, Computar, NC) by a high speed CMOS camera (Basler, Ace aca 2000-340 km, Germany). The iris in the imaging lens adjusted the pixel to speckle ratio. The linear polarizer, in front of the imaging lens, allowed capturing speckle images at both parallel and cross polarized states^15^. Cross-polarized collection minimized the specular reflections, providing fully developed, high-contrast speckle patterns. Additional co-polarized acquisition enabled assessing the average size of scattering centers^15^. The speckle frames were acquired at 739 fps, over an RoI of 510 × 510 pixels, for at least 5 seconds, and transferred to the computer via CameraLink interface.

To evaluate the G^*^(*ω*), speckle frames were processed as outlined in the flowchart of Fig. 2. Briefly, cross-correlating the first speckle frame with subsequent frames returned the speckle intensity auto-correlation function, *g*_*2*_(*t*) (Fig. 2, box 2)^8^,^9^,^11^,^12^,^13^. Spatial averaging over the entire ROI and temporal averaging of multiple evolving *g*_*2*_(*t*) curves provided statistical accuracy^8^,^9^,^10^,^11^,^12^,^13^,^16^. Temporal averaging of speckle frames provided the DRP at both parallel and cross polarization states (Fig. 2, box 3). Subsequently, *μ*_*s*_′ was experimentally calculated by fitting a model derived from diffusion theory to the radial cross-polarized DRP (Fig. 2, Box 4)^13^,^15^,^16^,^31^. The *μ*_*s*_′ determined the *γ* and *ζ* parameters in the modified equation, *g*_*2*_(*t*) = *exp*(*−2γ (k*^*2*^ < *Δr*^*2*^(*t*)*>*)^*ζ*^) used to deduce the MSD^13^,^15^ (Fig. 2, Box 5). Meanwhile, the ratio of parallel-polarized DRP along short and long axes, i.e. *Î* = *I*(*φ* = *90*^*o*^)*/I*(*φ* = *0*^*o*^), was compared with a calibration curve to evaluate the average radius of scattering particles, *a* (Fig. 2, Box 6). Finally, *MSD* and *a* were substituted in GSER to calculate the G^*^(*ω*) (Fig. 2, Box 7).

To evaluate the spatially-resolved 2D map of G^*^(*ω*) in the micro-fabricated phantom, the focusing lens was removed from the setup to enable expanded beam illumination. Moreover, imaging lens was replaced with an objective (10×, Olympus) and a tube lens (focal length 175 mm) to acquire high-resolution, magnified speckle images. The FOV covered a 500 × 500 μm^2^ area, i.e. 1000 × 1000 pixels. The phantom was mounted on a translational stage and scanned at 450 μm steps. At each scanning location, speckle images were acquired at 250 fps for 1 second. Spatially-resolved *g*_*2*_(*t*) curves for individual pixel of the frame series were obtained by limiting the spatial averaging to a 25 × 25 Gaussian window of neighboring pixels. Since expanded illumination was not conducive to calculating the *μ*_*s*_′ and *a*, conventional equation, i. e., *g*_*2*_(*t*) = *exp*(*−3.3 (k*^*2*^ < *Δr*^*2*^(*t*)*>*)^*0.5*^), was used to deduce the *MSD* for individual pixels. Subsequently, spatially-resolved G^*^(*ω*) values were calculated by replacing the *MSD* and known *a* values in Eqn (1).

### Mechanical rheometry

The G^*^(*ω*) of hydrogels were measured using a strain-controlled AR-G2 rheometer (TA Instruments, DE). The PEGDA gels were placed on the bottom plate. An 8 mm. dia. top plate was lowered in 50 μm steps, while monitoring the applied normal force, until the sample was securely sandwiched between the two plates. A frequency-sweep procedure was conducted, at 0.1% strain and 25 °C, to obtain the viscoelastic modulus, G*, at 0.1–100 Hz frequencies. Similarly, for highly viscoelastic agarose and PA gels, 8 mm dia. disks were punched out of the gels and evaluated as above. For softer agarose 0.5–1.5%, and (3%-A, 1%-B) PA, (A7.5%, B0.05%), and (A7.5%, B0.2%) hydrogels, cutting the samples to 8 mm disks were impractical. Moreover, the small contact area of an 8 mm plate limited the upper frequency range for these softer samples. Instead, a 40 mm dia. top plate was employed and the precursor solutions of the gels were directly pipetted on the bottom plate and sandwiched between the two plates. Moisture traps were placed to prevent drying. Repeated frequency sweeps were conducted every 30 minutes until no further growth was observed in the evaluated moduli, indicating that the gel has fully cured.

### AFM-based micro-indentation

The indentation moduli of hydrogels were quantified using an Asylum MFP3D atomic force microscope (Asylum Research, CA). We used polystyrene colloidal probe tips with radius *R* ∼ 12.5 *μ*m (Polysciences, PA) attached to tip-less cantilevers with nominal spring constants of *k* ∼ 0.12 N/m (Bruker, CA). The colloidal probes were attached to the cantilever via lift-off process^39^. For each probe tip, the exact spring constants of the cantilevers were directly measured using thermal calibration method^58^. The relationship between the detected voltage and the applied force was calibrated by bringing the cantilever in contact with a glass slide and calculating the slope of the voltage-displacement curve. The displacement, *d*, was translated to force, *F*, using Hooke’s Law (*F* = *kd*). The indentation was performed under a force control scheme (max force ~20 nN), limiting the indentation depths to 0.5–3 μm. Each hydrogel was indented at 8–10 locations with 100 um spacing. The Hertz model was fit to the force-displacement curve to obtain the indentation modulus, *E*. An indentation velocity of 2 μm/s ensured evaluating the indentation modulus at close to equilibrium condition.

## Additional Information

**How to cite this article**: Hajjarian, Z. *et al*. Laser Speckle Rheology for evaluating the viscoelastic properties of hydrogel scaffolds. *Sci. Rep.* **6**, 37949; doi: 10.1038/srep37949 (2016).

**Publisher's note:** Springer Nature remains neutral with regard to jurisdictional claims in published maps and institutional affiliations.

## Supplementary Material

Supplementary Information

Supplementary Video 1
