# Supplementary material for: Laser Speckle Rheology for evaluating the viscoelastic properties of hydrogel scaffolds
Source: Sci Rep. 2016 Dec 1;6:37949. doi: 10.1038/srep37949 (PMC5131361; doi:10.1038/srep37949)
Supplement: Supplementary Information [file srep37949-s1.pdf]

Laser Speckle Rheology for evaluating the viscoelastic properties of hydrogel scaffolds

Zeinab Hajjarian[1], Hadi Tavakoli Nia[2], Shawn Ahn[3], Alan J. Grodzinsky[4-6], Rakesh K. Jain[2], and Seemantini K. Nadkarni[1*]

[1]Wellman Center for Photomedicine, Massachusetts General Hospital, Harvard Medical School, Boston, MA

[2]Edwin Steele Laboratory for Tumor Biology, Massachusetts General Hospital, Harvard Medical School, Boston, MA

[3]Department of Electrical and Computer Engineering, University of Illinois at Urbana-Champaign, Urbana, IL

[4]Department of Mechanical Engineering, Massachusetts Institute of Technology, Cambridge, MA

[5]Department of Biological Engineering, Massachusetts Institute of Technology, Cambridge, MA

[6]Department of Electrical Engineering, Massachusetts Institute of Technology, Cambridge, MA

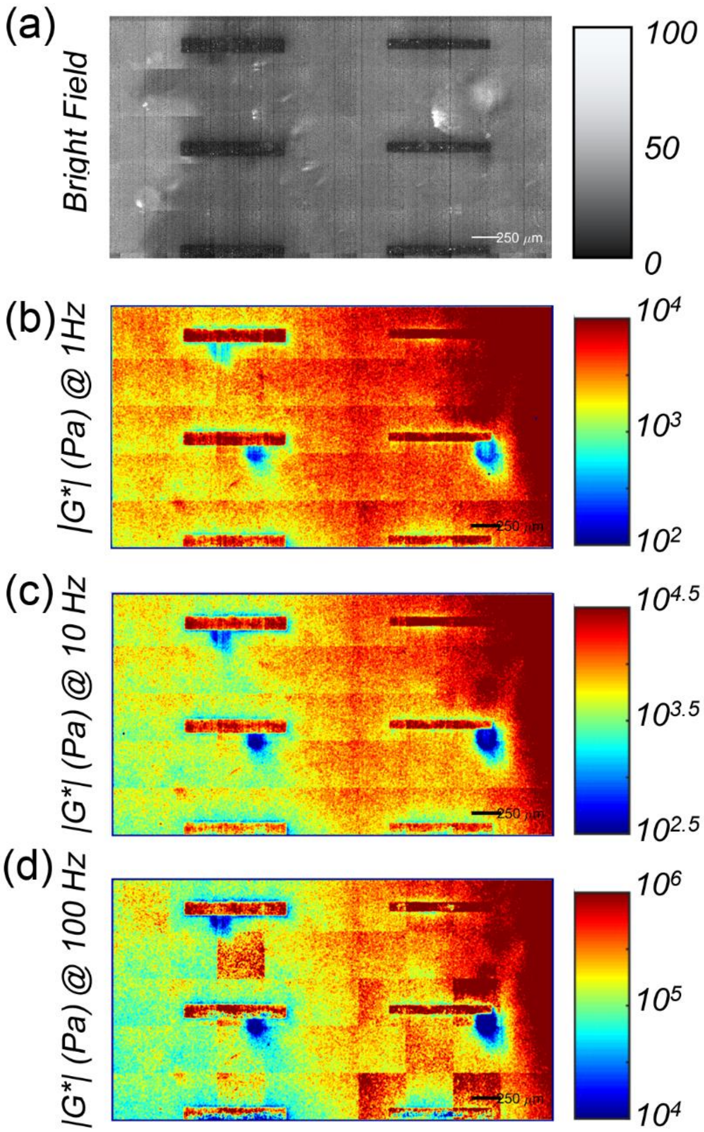

Supplementary Figure S1. (a) Bright field image of the micro-fabricated composite PDMS-PEGDA 10% phantom. A total of 6 PDMS bars are visible within the PEGDA background. The bars in successive columns are 1 mm long and 100, and 80 µm wide, respectively. **(b)** Spatially-resolved G*, evaluated at 1 Hz. In the color-bar, the moduli range of 100 Pa - 10 kPa, are represented by blue to red hues. **(c)** Spatially-resolved G*, evaluated at 10 Hz. In the color-bar, the moduli range of 300 Pa - 300 kPa, are represented by blue to red hues. **(d)** Spatially-resolved G*, evaluated at 100 Hz. In the color-bar, the moduli range of 10 kPa - 1 MPa, are represented by blue to red hues. Compared to Fig. 7, the contrast between PDMS bars and the PEGDA 10% background is reduced at all length-scales and frequencies. Moreover, higher stiffness of the gel constituents prevent leakage between various compartments and leads to more defined borders and increased homogeneity of moduli within the bars.

Supplementary Video Legend

Supplementary Video V1. Spatially-resolved frequency-dependent viscoelastic modulus of the micro-fabricated, composite PDMS-PEGDA gel phantom. The frames of this video file represent the spatially-resolved G* at several consecutive oscillation frequencies in ω=0.3-100 Hz range. The bars in successive columns are 1 mm long and 200, 150, 100, and 80 μm wide, respectively. The color-bar scale is adjusted at each frame to provide the highest visual contrast between the soft PEGDA background and the stiff PDMS bars. The 80 μm wide stiff PDMS bars evolve from barely visible at low frequencies to fully resolved and highly contrasted from the soft PEGDA background at higher frequencies within the G^* color maps.
